# The role of fundamental movement skills on children’s physical activity during different segments of the school day

**DOI:** 10.1186/s12889-024-18769-3

**Published:** 2024-05-10

**Authors:** Dongao Liu, Zan Huang, Yanjie Liu, Yulan Zhou

**Affiliations:** 1 Physical Education Department, Shanghai University of Finance and Economics, Shanghai, 200433 China; 2 College of Physical Education and Health Sciences, Zhejiang Normal University, Jinhua City, Zhejiang Province 321004 China

**Keywords:** Physical activity, Children, Segments, Object-control, Locomotor

## Abstract

**Background:**

Although prior studies have demonstrated that children with high levels of fundamental movement skill (FMS) are more active throughout the day, little is known about children’s FMS and their physical activity (PA) during different segments of the school day (e.g., recess, lunch break, and physical education). The present study focused on FMS and moderate-to-vigorous PA (MVPA) during school day and identifies the association between children’s FMS and MVPA during different segments of the school day in China.

**Methods:**

A total of 322 children (boys *n* = 163, girls *n* = 159; M_age_ = 8.12, SD = 1.22 years) from four elementary schools involved in this study. Children’s FMS and MVPA were measured using the Test of Gross Motor Development-2nd edition (TGMD-2) and hip-mounted accelerometers. Data such as height, weight, and socio-economic status (SES) were also obtained. Multilevel mixed regression models were used to examine the cross-sectional associations between FMS and MVPA. Models were adjusted for gender, age, standardized body mass index, and SES.

**Results:**

Children engaged in 32.19 min of MVPA during the whole school day. Boys were more active than girls and had higher object-control skills competency. Locomotor skills were positively associated with children’s long recess (B = 1.063) and short recess time (B = 1.502) MVPA. Object-control skills were positively correlated with children’s MVPA time during long recess (B = 1.244) and physical education (PE) lessons (B = 1.171).

**Conclusion:**

The findings highlight the importance of developing both locomotor and object-control skills in elementary schools to lead more MVPA engagement during different segments of the school day.

## Background

Moderate-to-vigorous physical activity (MVPA) has been identified with many health benefits, such as enhancing muscular strength, improving cardiovascular function, metabolic syndrome, and mental wellbeing [1]. The World Health Organization recommends that children and adolescents should engage in at least 60 min of MVPA daily [2]. However, Chinese children and adolescents were found to be physically inactive [3–5]. Liu et al. investigated MVPA time of 125,281 children and adolescents from 31 provinces in China and found that only 13.1% of them meet this requirement [4]. Wang et al. used accelerometers to measure the MVPA of 2,163 students in the 4th to 11th grade from 11 cities in China and showed children and adolescents spent an average of 28.26 min/day engaged in MVPA [5].

Schools are identified as a key setting for children to be physical active, as they spend a considerable proportion of their time within the school environment [6]. In-school time periods include different segments such as physical education (PE), lunch break, and recess time allowing children the opportunity to be physical active. PE lessons represent an important source of daily MVPA obtained during school time and has the potential to contribute up to 21–28% of children’s daily MVPA recommendations [7, 8]. Lunch break and recess time are also critical for children to be active on a daily basis. These periods can contribute to 5–40% of their daily MVPA [9–12]. This emphasizes the need to identify potential factors that are associated with children’s MVPA during different segments of the school day for enhancing their daily PA levels.

Fundamental movement skills (FMS) are considered as the building blocks for more complex motor skills and provide the foundation for participation in many forms of PA [13]. FMS are typically classified into three areas: locomotor (e.g., running, jumping), object manipulation (e.g., catching, throwing), and stability skills (e.g., balance) [14]. A systematic review shows global levels of FMS are generally below to average [15]. It is suggested that higher levels of FMS competency will provide greater opportunities for children to engage in PA at different segments of the school day [16–18]. Gu et al. investigated the association between FMS and children’s MVPA during PE lessons in the United States and found that FMS was a significant predictor of children’s MVPA [16]. The study conducted in Brazil focused on the relationship between locomotor and object control skills and children’s MVPA during PE lessons and revealed that while object-control skills were positively associated with six to eight years children’s MVPA, locomotor skills were not [17]. Cohen et al. examined the associations between FMS and MVPA during the school day (lunchtime and recess) among children attending primary schools in Australia. The study showed that similarly, object-control skills were positively associated with children’s MVPA during lunchtime and recess period, the same still could not be said for locomotor skills [18].

While these studies investigated the association between FMS and children’s MVPA during different segments of the school day in United States, Brazil, and Australia, the extension of these findings to other counties (such as China) remain unclear. Furthermore, within structured settings like PE lessons, where students engage in organized activities under the guidance of a PE teacher, the influence of FMS on children’s MVPA may vary compared to controlled conditions such as recess periods, where students participate in supervised activities led by classroom teachers without direct interaction. Additionally, in free play conditions such as lunch breaks, where students have the freedom to choose whether to be active or not, the influence of FMS on children’s MVPA might show unique variations [12, 19]. To the authors’ knowledge, the influence of FMS in relation to children’s MVPA during the whole school day is yet to be explored. Insights on the impact of FMS on MVPA at specific context during school day could help make future recommendations for more targeted PA interventions. Therefore, this study seeks to examine the association between FMS and children’s MVPA throughout the school day in China.

## Methods

### Participants

The research procedures were approved by the ethics committee of the University and other relevant school authorities. Four public elementary schools situated in the Wucheng and Jindong districts of Jinhua, Zhejiang, a city in southern China, were selected using a convenience sampling approach. All public primary schools in Jinhua fall under the unified management of the local Education Bureau, overseeing aspects such as school facilities, enrollment procedures, class size, and timetables. Two classes were randomly sampled from each grade level (from First to Fourth Grade) in every school. The average class size ranged from 36 to 46 children. A total of 334 children aged six to ten years from eight classes were invited to participate. Of the 334 children, parental written informed consent and child verbal assent were obtained for 328 children (98.2%) prior to data collection. However, out of the 328 participants, six children were excluded because their valid accelerometer data did not cover at least three school days. Therefore, 322 participants whose data on FMS and MVPA were available were included in the final analysis.

### Settings

The regular school day starts at 8:10 a.m. for all grade levels and ends at 3:15 p.m. for Grades One to Two, and 4:00 p.m. for Grades Three to Four. Thus, the during-school period is defined as 8:10 a.m. to 3:15 p.m. for Grades One to Two, and 8:10 a.m. to 4:00 p.m. for Grades Three to Four.

Time segments during the school period were defined as lunch break, recess, and PE lessons. Lunch break lasts from 12:05 p.m. to 13:30 p.m., which includes lunchtime, break, and reading and writing. Lunchtime is offered in the classroom and lasts 20 min. After lunchtime, children take a short break (25 min) and are then provided with a 40-minute reading and writing time. Recess comprises two long parts (20- and 30-minutes) and four or five short ones (10 min each segment). During every long recess (i.e., 20–30 min), all children compulsory walk to the playground/outdoor spaces and use the equipment provided by the school, such as sports balls and jump rope to practice. The classroom teachers supervised children’s safety, but they did not interact with children to encourage them to be active. During the short recesses, children transition from classroom to sports field and are free to perform activities, such as chasing games, running, and sitting. No unfixed equipment (e.g., balls, hoops) is unavailable for children during lunch break and short recess time in all four schools. In this study, long and short recess break are treated as separate entities due to their distinct characteristics (e.g., available resources, space, and supervision). Therefore, the total long recess time for each Grade is 50 min. The total short recess time are 40 and 50 min for Grade One-Two and Grade Three-Four, respectively. The specific information about the recess periods is shown in Table 1.

**Table 1:** Recess periods of the elementary school

	Time	Minutes		Main contents
		Grade One-Two	Grade Three-Four	
1	8:10 − 8:30	20	20	Long recess
2	9:10 − 9:40	30	30	Long recess
3	10:20 − 10:30	10	10	Between the second and third lesson
4	11:10–11:20	10	10	Between the third and the fourth lesson
5	13:30 − 13:40	10	10	Between lunch and the fifth lesson
6	14:20 − 14:30	10	10	Between the fifth and the sixth lesson
7	15:15–15:25	----	10	Between the sixth and the seventh lesson
	Total long recess	50	50	----
	Total short recess	40	50	----

According to the school’s overall schedule, the participating schools provide four PE lessons per week for Grades One and Two, and three PE lessons per week for Grades Three and Four. The PE lessons in these elementary schools are co-educational and taught by certified PE teachers. The main content areas of the PE curriculum are either basketball, football, or volleyball and aims to improve children’s sports skills. A typical 40-minutes PE lesson comprises three routine sessions: (a) lesson introduction and warm-up led by the PE teacher (5 min); (b) skill instruction and practice (30 min). Teachers demonstrate and explain a sport’s skill (e.g., passing a basketball, kicking the football, or volleyball serving), and instruct students to learn and practice; and (c) cool-down and conclusion (5 min).

### Variables and measures

#### Fundamental movement skills

The study used the Test of Gross Motor Development-2nd edition (TGMD-2) to assess children’s FMS. The TGMD-2 is widely acknowledged as a robust process-oriented tool for assessing individual skills through the demonstration of specific movement components. It has demonstrated strong validity and reliability, particularly for children with typical development between the ages of 3 and 10 [20, 21]. The TGMD-2 includes two subscales: locomotor (i.e., running, galloping, leaping, horizontal jumping, sliding, and hopping) and object-control (i.e., striking, dribbling, rolling, throwing, catching, and kicking) skills. For each skill, a child was evaluated on 3 to 5 performance criteria with a “1” scored if the criterion was present and a “0” scored if the criterion was absent [20]. The combination of all skill raw scores were summed to give a total FMS score, while locomotor and object-control subtest scores were created by totaling the scores of skills within each subscale.

#### Physical activity

Children’s MVPA during school were measured using Actigraph wGT3X-BT accelerometers, which are valid and reliable devices used to measure MVPA in school conditions [22]. The research assistants demonstrated how to wear accelerometers, and instructed each child to use an elastic belt to fasten the accelerometer to their right hipbone for five entire school days [11]. Accelerometers may be retained except for water-based activities (e.g., bathing or swimming). ActiLife 6.5 software was used to analyze the data. Only children recording more than five hours of accelerometer data (excluding strings of zeros for 20 min or longer) on at least three school days (including one PE day) were used in the analysis [23]. The sampling interval in the present study was set at 15-s epoch with a frequency of 30 Hz [24]. Raw activity counts were interpreted using the cut-off points for Chinese children, which defined different intensities of PA (MPA: 2800–3999; VPA: ≥4000 counts per minute) [25]. The data for each child were truncated and then matched with the original time frames for each segment of the school day. The average time (minutes) spent in MVPA on various segments of all valid school day was reported.

#### Demographics data

The participants’ gender, date of birth, and family incomes were obtained from the parent questionnaire. Parents reported their and their spouses’ incomes. According to Zhejiang’s per capita disposable income, the family annual income was categorized as having either lower or higher socio-economic status (SES).

#### Height and weight

Height and weight were measured without shoes and heavy clothing. A portable stadiometer was used to measure standing height with the value recorded to the nearest millimeter. Weight was measured using an electronic calibrated scale, with weight recorded to the nearest 0.1 kg (kg). For each child, the body mass index (BMI) was calculated from weight and height values, using the formula: weight (kg)/height (m)^2^. BMI z-scores were calculated on the basis of the World Health Organization growth standards [26].

### Procedure

Data was collected by one author and four trained research assistants (graduate students majoring in sports pedagogy) during the months of March to April 2022 (eight weeks), with each school scheduled for two weeks. The FMS, BMI, and demographics data assessments were completed in the first week. The children’s MVPA during the school day was then measured by research assistants during the second week. The children’s FMS were assessed in small groups of three to four using TGMD-2. Children received instructions and demonstrations of the skill before performance, and were then asked to complete two trials of each skill. Four trained research assistants (trained by a professor with experience in evaluating the TGMD-2) implemented and video-recorded the TGMD-2 with small groups of children. Then research assistants worked in pairs to analyze the videos. Approximately 20% of the videos were randomly selected for a reliability check. The mean agreement between the two research assistants were 0.93 and 0.95 for the locomotor and object control subscales, respectively, which was deemed acceptable [27]. During the MVPA measurement days, upon arrival to the school, children were provided with a specific numbered accelerometer to wear and remove at the end of the school day. Children were provided with the same accelerometer for the next day. The research assistants monitored the children to ensure that the accelerometers remained fastened during the entire school day. To ascertain whether the school timetabled break times adhered to the local education bureau, the researchers randomly checked each school’s time schedule for two days during the data collection period.

### Data analysis

All analyses were performed using IBM SPSS Statistics Version 26.0 and *P* < 0.05 was the set significant value. Prior to analysis, skew and kurtosis were computed to assess the normality of all variables. Variables that did not follow a normal distribution were log-transformed to achieve a more normal distribution. Therefore, total daily, short, and long recess MVPA minutes were log-transformed in this process. Descriptive statistics (e.g., mean, SD, and frequencies) were used to describe children’s demographics characteristics, the FMS scores, and the MVPA minutes. Gender differences in children’s demographics, FMS, and MVPA during school days were tested using analysis of variance (ANOVA). Partial eta-squared (η^2^) was used as the effect size for ANOVA. The values of 0.01, 0.06, and 0.14 were designated as small, medium, and large effect sizes, respectively [28]. Multilevel mixed linear regression models were performed to assess the associations between FMS (locomotor or object-control skills) and MVPA time on long recess, short recess, lunch break, and PE lessons. The different segments of MVPA time during school day entered as the outcome variables, FMS (i.e., locomotor or object-control skills) as the predictor variable, and school as a random factor. All models were adjusted for gender, age, BMI z-score, and SES [29, 30].

## Results

### Participant characteristics

The summary data and gender differences for children’s demographics characteristics, FMS, and MVPA are presented in Table 2. The final study population consisted of 322 children, including 163 boys and 159 girls, with a mean age of 8.12 ± 1.22 years. Children’s average BMI z-score was 0.44 (SD = 1.14), and 86.0% of the sample was categorized as high SES. The BMI z-score exhibited a significant gender disparity [F (1, 320) = 5.995, *P* = 0.015, η^2^ = 0.039], with boys presenting a higher z-score. Participating children’s mean scores for FMS was 65.29 (SD = 8.17) with 33.43 (SD = 3.97) for locomotor skills and 31.86 (SD = 4.75) for object control-skills. There was a gender difference in object-control raw scores favoring boys [F (1, 320) = 24.367, *P* = 0.000, η^2^ = 0.071], but not in locomotor raw scores [F (1, 320) = 1.116, *P* = 0.291, η^2^ = 0.003]. Children spent an average of 32.19 ± 13.04 min in MVPA during the school day, which corresponds to a mean of 7.19% (*SD* = 2.91) of school-time day. An ANOVA revealed the significant gender differences on the MVPA time of children during the total school day [F (1, 320) = 25.283, *P* = 0.000, η^2^ = 0.073], short [F (1, 320) = 32.500, *P* = 0.000, η^2^ = 0.093] and long recess [F (1, 320) = 11.688, *P* = 0.001, η^2^ = 0.035]. Compared with girls, boys accumulated more MVPA time during those segments. There were non-significant differences between boys and girls with respect to MVPA during lunch break [F (1, 320) = 2.685, *P* = 0.052, η^2^ = 0.024] and PE lessons [F (1, 320) = 2.538, *P* = 0.061, η^2^ = 0.031].

**Table 2:** Descriptive and gender differences for children’s background, fundamental movement skills and physical activity

	All (*N* = 322)		Boys (*N* = 163)		Girls (*N* = 159)		Gender difference
	Mean	SD	Mean	SD	Mean	SD	*P* Value
Background							
Age	8.12	1.22	8.12	1.30	8.13	1.14	0.982
BMI z-score	0.44	1.14	0.60	1.28	0.29	0.95	0.015^*^
SES ^a^							
Low SES	45	14.0	22	13.5	23	14.5	—
High SES	277	86.0	141	86.5	136	85.5	
FMS							
Locomotor skills	33.43	3.97	33.66	3.97	33.20	3.97	0.291
Object-control skills	31.86	4.75	33.10	3.89	30.58	5.20	0.000^*^
FMS total	65.29	8.17	66.76	7.44	63.77	8.63	0.001^*^
Physical activity							
Total daily MVPA minutes	32.19	13.04	35.87	13.56	28.42	11.35	0.000^*^
Total daily MVPA%	7.19	2.91	8.02	3.03	6.35	2.54	
Short recess MVPA minutes	5.24	3.74	6.18	3.97	4.43	3.05	0.000^*^
Short recess MVPA%	1.17	0.84	1.38	0.89	0.99	0.68	
Long recess MVPA minutes	8.09	2.46	9.10	2.48	7.07	2.34	0.001^*^
Long recess MVPA%	1.81	0.55	2.03	0.55	1.58	0.52	
Lunch break MVPA minutes	2.94	1.88	3.02	0.46	2.84	0.58	0.052
Lunch break MVPA%	0.66	0.42	0.67	0.10	0.63	0.13	
PE MVPA minutes	15.92	3.27	16.43	2.50	15.38	2.69	0.061
PE MVPA%	3.56	0.73	3.67	0.56	3.44	0.60	

Abbreviations: BMI, Body mass index; SES, Socioeconomic status; FMS, Fundamental movement skills; MVPA, Moderate-to-vigorous physical activity; PE, Physical education

^a^ Data are presented as number (%)

*, *P* < 0.05

### Locomotor skills and MVPA

Table 3 presents the association among children’s locomotor skills and their MVPA during different segment of the school day. After adjustment for gender, age, BMI-z score, and SES, the findings demonstrated a positive association between locomotor skills and the duration of MVPA during long (B = 1.063, *P* = 0.008) and short recess periods (B = 1.502, *P* = 0.037). However, no significant relationship was observed between locomotor skills and MVPA during children’s lunch break (B = 0.070, *P* = 0.195) and PE lessons (B = 0.340, *P* = 0.076).

**Table 3:** Summary of mixed regression analyses for locomotor skills and MVPA minutes

	B	SE	LCI	UCI	*P*
Model 1: Long recess MVPA					
Gender ^b^	0.902	0.256	0.398	1.405	0.000
Age	0.781	0.782	−0.758	2.319	0.319
BMI z-score	−0.096	0.112	−0.316	0.123	0.388
SES	−0.146	0.361	−0.856	0.565	0.687
Locomotor skills	1.063	0.250	0.032	1.157	0.008
Model 2: Short recess MVPA					
Gender ^b^	2.414	0.525	1.726	3.101	0.000
Age	1.235	0.215	1.073	2.562	0.021
BMI z-score	−0.074	0.152	−0.374	0.225	0.625
SES	0.457	0.493	−0.513	1.428	0.354
Locomotor skills	1.502	0.095	1.008	2.253	0.037
Model 3: Lunch break MVPA					
Gender ^b^	1.091	0.284	0.532	1.650	0.000
Age	−0.559	1.001	−2.537	1.419	0.579
BMI z-score	−0.093	0.124	−0.337	0.150	0.453
SES	0.523	0.401	−0.265	1.312	0.193
Locomotor skills	0.070	0.054	−0.036	0.177	0.195
Model 4: PE lesson MVPA					
Gender ^b^	2.797	0.672	1.475	4.118	0.000
Age	4.461	1.778	0.962	7.960	0.016
BMI z-score	0.188	0.292	−0.387	0.764	0.520
SES	0.879	0.947	−0.985	2.743	0.354
Locomotor skills	0.340	0.128	−0.088	0.592	0.076

Note: B, beta; SE B, standard error beta; 95% CI, confidence interval; L, lower; U, upper; MVPA, time spent in moderate-to-vigorous PA.

^b^ Reference category is girl

### Object-control skills and MVPA

Table 4 demonstrates the relationship among children’s object-control skills and their MVPA during different segments of the school day. When adjustment for gender, age, BMI-z score, and SES, the analysis indicated a positive link between object-control skills and the amount of MVPA during long recess (B = 1.244, *P* = 0.007) and PE lessons (B = 1.171, *P* = 0.014). Nevertheless, no significant correlation was found between object-control skills and MVPA during children’s short recess (B = 0.053, *P* = 0.345) and lunch break (B = 0.034, *P* = 0.187).

**Table 4:** Summary of mixed regression analyses for object-control skills and MVPA minutes

	B	SE	LCI	UCI	*P*
Model 1: Long recess MVPA					
Gender ^b^	0.952	0.281	0.399	1.505	0.001
Age	1.113	0.909	−0.675	2.902	0.221
BMI z-score	−0.119	0.111	−0.337	0.099	0.285
SES	−0.152	0.363	−0.866	0.561	0.675
Object-control skills	1.244	0.222	0.781	1.851	0.007
Model 2: Short recess MVPA					
Gender ^b^	2.291	0.378	1.547	3.034	0.000
Age	1.216	0.309	0.392	1.824	0.031
BMI z-score	−0.083	0.151	−0.379	0.213	0.581
SES	0.479	0.493	−0.490	1.449	0.331
Object-control skills	0.053	0.056	−0.057	0.163	0.345
Model 3: Lunch break MVPA					
Gender ^b^	1.051	0.312	0.438	1.665	0.001
Age	0.214	0.744	−1.250	1.677	0.774
BMI z-score	−0.111	0.123	−0.352	0.131	0.369
SES	0.532	0.402	−0.259	1.324	0.187
Object-control skills	0.034	0.457	−0.259	1.324	0.187
Model 4: PE lesson MVPA					
Gender ^b^	2.130	0.726	0.701	3.558	0.004
Age	3.887	0.601	0.295	6.070	0.023
BMI z-score	0.191	0.289	−0.377	0.760	0.508
SES	0.911	0.945	−0.949	2.771	0.336
Object-control skills	1.171	0.107	0.053	1.476	0.014

Note: B, beta; SE B, standard error beta; 95% CI, confidence interval; L, lower; U, upper; MVPA, time spent in moderate-to-vigorous PA.

^b^ Reference category is girl

## Discussion

The study aims to examine the associations between FMS and children’s MVPA during time periods of the school day that represent important PA opportunities for children. It was found that locomotor skill competency was positively associated with children’s MVPA during long and short recess time at school. Object-control skill competency was positively associated with MVPA during long recess and PE lessons at school. Children spent 7.19% of their school-time on MVPA, and boys accumulated more minutes of MVPA than girls during the total school day, and short and long recess. Children’s FMS score was 65.29, and boys had better object-control skills than girls.

In the present study, short recess and lunch break perioded were considered as opportunities for free play, where children engage in PA spontaneously without a prescribed routine or specific purpose. On the one hand, children’s MVPA during short recess time was positively associated with locomotor skills, but not with object-control skills. Similarly, Tsuda et al. found children’s locomotor skills were associated with MVPA during free-play time at school [12]. In contrast, the study conducted by Cohen et al. found object-control skills, but not locomotor skills, were significantly associated with MVPA during recess time [18]. The inconsistencies among existing literature suggest that the relationship between skill type and MVPA during recess time may influenced by a range of contextual factors—activities and equipment. Children accrue MVPA through informal play-like (e.g., running and chasing games) and jumping activity types during short recess time in current study, which require a high level of locomotor rather than object-control skills [31]. Furthermore, available school equipment during recess may also influence the skill type predicting children’s MVPA [9]. In line with Greece and other countries [32, 33], children in China had limited or no access to object-control equipment (e.g., balls) during short recess time, potentially constraining their ability to engage in activities such as soccer and basketball that require proficient object-control skills. Hence, there was no significant association between object-control skills and children’s MVPA during short recess time.

On the other hand, the results of the regression analyses indicated that both locomotor and object-control skills were not identified as predictors of children’s MVPA during lunch break. This finding veer away from the study conducted by Cohen et al. which reported object-control skills were positively associated with lunchtime MVPA [18]. Evidently, FMS can enhance physically active play opportunities for children [12, 34, 35]. The absence of a significant link between FMS and MVPA during the lunch break in this study raises uncertainties. It is plausible that the restricted movement opportunities available to children during this time period limited the impact of FMS on their levels of PA. In accordance with Chinese tradition, lunch breaks are typically seen as a time for sedentary pursuits like sitting, reading, writing, napping, and socializing with friends [11]. This restrictive mindset hinders children from enjoying active play during their lunch break [36]. Compared with the study conducted by Cohen et al., the MVPA time of children during lunch break in present study was shorter (5.4 min vs. 2.9 min) [18]. It is crucial to change the existing mindset and adopt effective strategies in order to promote children’s engagement in MVPA during lunch breaks.

The current study found that both locomotor and object-control skills were positively associated with long recess MVPA. The finding suggests that the majority of MVPA during long recess is a result of the controlled setting provided by the school, where children are expected to follow a daily routine of PA under the supervision of their classroom teacher. During this condition, all children compulsory to participate in the activities prescribed by the school and are provided with the necessary equipment. Activities such as basketball, football, and jump rope, known for their high levels of physical engagement and reliance on locomotor and object-control skills. It is possible that more skilled children gravitate towards these games and utilize a larger portion of the available activity areas, resulting in elevated levels of MVPA [37]. In addition, classroom teachers’ supervision during the long recess may allow children with high FMS to show off and demonstrate their skills for their teachers, thereby engaging in more MVPA [38]. Since children accumulate the second-highest amount of MVPA during long recess breaks (8.09 min), following PE lessons (15.92 min), it is necessary to implement strategies that effectively promote children’s participation in MVPA during these extended recess periods.

The positive correlation between children’s object-control skills and their MVPA in structured PE lessons indicates that children with higher levels of competence in object-control skills are more inclined to participate in greater amounts of MVPA during these classes. Unsurprisingly, this is because the main content areas of PE curriculum in this study is sports skills practice (e.g., passing a basketball, kicking the football, or volleyball serving). Being able to perform object control skills, such as the ability to catch or throw in basketball, may give children more opportunities to play games and be active [35]. Additionally, previous research found that at different ages, the motor demands for PA during PE lessons may change. Object-control skills were positively associated with the 6–8 years age group children’s PA, whereas locomotor and object-control skills were related to PA for children aged 9–10 [17]. Further longitudinal studies are necessary to understand the association between FMS and children’s MVPA during PE classes across childhood.

Children typically participated in MVPA for 32.19 min throughout the school day, accounting for approximately 7.19% of the total school day. The result aligns with previous research conducted on children in China and Europe within the same age range. Those studies consistently showed that children allocate only a minimal portion of their school hours to MVPA (5–16%), failing to fulfill the WHO’s recommended 60 min daily activity target [11, 39, 40]. Safety concerns and a lack of accessible equipment in schools can both limit opportunities for PA, contributing to lower levels of MVPA among Chinese children during the school day [11]. Given the increasing time Chinese children dedicate to academic pursuits to enhance their performance, there is limited availability for participating in PA outside of school [41]. The school should be identified as an important setting for children’s PA promotion, and is necessary to develop intervention strategies to promote children’s MVPA in school-time to contribute to their daily PA levels. Mirroring previous studies, the results of this study showed that boys accumulated more minutes of MVPA than girls during the total school day, and long and short recess [18, 23, 39, 42]. Evidence also indicates that boys are more active than girls in both school days and under other contexts such as before school, after school, and weekends [43, 44]. The perception of gender roles among children may influence their engagement in MVPA [45]. Researchers argue that societal expectations and early socialization often lead boys and girls to adopt different behaviors and participate in activities that align with their gender norms [46]. Boys tend to be encouraged to engage in high-intensity activities like basketball, football, and chasing. In contrast, girls often participate in low-intensity activities such as walking, dancing, and aerobics [47]. Thus, boys accumulated more MVPA time than girls. This finding implies that more attention and targeted intervention should be considered to increase the MVPA time of girls.

In this study, the average FMS score for children was 65.29 (SD = 8.17). A systematic review conducted by Bolger et al. highlights significant variations in FMS scores among children aged 6–10 years across different regions (such as Asia, Europe, and South America) when utilizing the same FMS measurement techniques [15]. For instance, Khodaverdi et al. found that children in Iran had an average FMS scores of 76.26 (SD = 9.28) [48], whereas children in the Czech Republic [49] and Chile [50] had FMS score of 87.68 (SD = 6.85) and 65.5 (SD = 8.6), respectively. The global FMS level of children aged 6–10 is currently considered “below average” compared to data collected in 1997–1998 [15]. This suggests that there is a need for more awareness-raising activities to address this issue. Schools, particularly PE lessons, are expected to provide an ideal setting for the development of children’s FMS. The importance of integrating FMS competence into the PE curriculum is evident in curriculum guidelines [51, 52]. However, there are calls to review whether the pedagogical approach to teaching PE is fit for this purpose [53]. In several countries, including China, Korean, and Portugal, many PE teachers adhere to a preferred model that emphasizes the specificity of motor skills. According to this model, motor skills are believed to require isolation and repetitive practice over an extended period of time [54, 55]. Advocates of reducing a skill to its component parts or phases of execution argue that by isolating and mastering each part separately, children can gradually reconstruct the skill as a whole [56]. Nevertheless, this approach may overlook the innate love for movement and intrinsic motivation that children possess, potentially reducing their chances to practice FMS [57]. Furthermore, teacher-led assessment has been widely acknowledged as a crucial element in supporting children’s development of FMS in PE. It provides teachers with valuable feedback to enhance learning standards and interventions [58]. However, the lack of sufficient professional development and training in PE for elementary generalist classroom teachers (e.g., the United Kingdom) [59], along with a limited understanding of appropriate assessment practices among specialist PE teachers (e.g., China) [60], can undermine their expertise and confidence in assessing motor skills effectively and hinder the development of children’s FMS [61]. It is crucial for PE teachers to adopt appropriate pedagogical strategies to enhance the quality of PE lessons. This includes integrating play-based and child-centered activities into the sports environment to encourage the practice of FMS and assess them in an optimal manner [62]. Moreover, differences between the gender were found in object-control skills, but not in locomotor skills. Consistently, boys scored higher in object-control skills than girls [12, 34]. Since boys tend to be more active and participate more in ball sport (e.g., football, tennis, and basketball) in China, it is possible that they get more opportunities to practice object-control skills [63]. It is important to recognize that children do not naturally develop FMS. These skills require intentional instruction and sufficient practice [64]. Hence, effective interventions should be designed according to the distinctive characteristics of FMS in boys and girls.

The current study has several strengths, including the use of a comprehensive qualitative assessment of movement characteristics of FMS, an objective measure of children’s MVPA during the school days, and control for the confounding factors in the analyses. Despite these strengths, some limitations should be considered. The TGMD-2 focus on isolated skill performance in closed or controlled environments, and subsequently are not reflective and assess the complex series of skills involved in sport and PA, which may limit their authenticity [65]. Future research may seek to use more effective FMS assessments. The participants in this study consist of children in grades 1 to 4 from four elementary schools in Zhejiang. It is crucial to recognize that this limited sample restricts the generalizability of the findings to other age groups and regions across China. Future research efforts should aim to include a more diverse sample encompassing a broader range of grade levels and geographical locations within China. Due to the cross-sectional design of the current study, there is limited research investigating the potential causal relationships between FMS competency and PA behavior. Future longitudinal and intervention studies are needed to determine the causal nature of the impact of FMS on children’s MVPA during different segments of the school day.

## Conclusion

The findings of present study extend the current literature by examining the role of FMS on children’s MVPA time during different segments of the school day in Zhejiang, China. Children who are more competent at locomotor skills engage in more MVPA during free play and controlled conditions (i.e., short and long recess time at school), and children who demonstrate more competent in object-control skills engage in more MVPA during controlled and structured settings (i.e., long recess and PE classes). Both locomotor and object-control skills are not significantly associated with children’s MVPA during lunch break. These findings suggest that targeted sports equipment and activities should be provided based on the relationship between different types of FMS and diverse activity conditions to promote MVPA in children during the school day. PE teachers can integrate movement opportunities into classroom routines (e.g., transitions, management) and instructional processes (e.g., running or jumping while passing the basketball). Children spent a meaningfully small proportion of lunch break time engaged in MVPA, which is of concern, and highlight the need to reassess school lunch break policies and to implement strategies aimed at maximizing children’s MVPA.

## Data Availability

The dataset analyzed during the current study are available from the corresponding author on reasonable request.

## Abbreviations

BMI: Body Mass Index
SES: Socioeconomic Status
TGMD-2: Test of Gross Motor Development-2nd edition
FMS: Fundamental Movement Skills
MVPA: Moderate-to-Vigorous Physical Activity
PE: Physical Education
